# Effect of a nitrogenous nanocomposite on leaching and N content in lettuce in soil columns

**DOI:** 10.1186/s11671-023-03874-w

**Published:** 2023-07-31

**Authors:** Ángel N. Rojas-Velázquez, Oscar I. Guillén-Castillo, Jorge A. Alcalá-Jauregui, Catarina Loredo-Osti, Hugo M. Ramírez-Tobías, Mauricio J. Romero-Méndez, Heriberto Méndez-Cortés, Alejandra Hernández-Montoya

**Affiliations:** Faculty of Agronomy and Veterinary Medicine, Autonomous University of San Luis Potosí, San Luis Potosí, Mexico

**Keywords:** Nitrogen, Nanofertilizers, Nitrate leaching, Bentonite, Organ clay, Nanocomposite

## Abstract

Nanofertilizers could promote nutrient efficiency with slow release compared to conventional fertilizers (CF). Most of the applied nitrogen is lost on the soil by leaching, due to the rapid release behavior of CF. Clays can function as a nanosized porous structure to retain and slowly release nutrients. The objective of this study was to evaluate a nitrogenous nanocomposite (NCN) and its effect on leaching and N content of lettuce (*Lactuca sativa*). The treatments applied were: 100% conventional fertilizer, 100% nitrogenous nanocomposite and the mixture in percentage of CF/NCN 25/75, 50/50, 75/25 and 25/0, 50/0 75/0% on columns of soil with lettuce for 45 days. Leachates at the end of the cycle increased in treatments with NCN. Treatments with NCN have higher N content in the leaf. In regard to biomass growth, leaf area, leaf N, drained variables, electrical conductivity and NO_3_^−^ content, it was possible to show that the doses of 50 and 75% of NCN match the characteristics of the crop compared to the control, which allows us to use lower doses than those recommended with CFs.

## Introduction

Conventional farming practices involve the extensive use of fertilizers that play a crucial role in achieving maximum yield [1]. However, due to the high solubility of conventional fertilizers, most nutrients are lost to the environment through surface runoff, denitrification, leaching and volatilization [2]. Faced with this situation, there are fertilizers that help to counteract these problems, and they are known as nanofertilizers, which have a function of slow release of nutrients, which improve the efficiency of nutrients and reduce the frequency of fertilization [3]. With the application of nanofertilizers in agriculture, sustainability in global food production could be achieved [4]. Nanofertilizers refer to macro- or micronutrient fertilizers, having a particle size of less than 100 nm that are used to promote crop productivity [5]. They can be grouped into three categories: The first is nanosupported fertilizers, materials with a nanometer structure that regulate the release of the fertilizer through adsorption processes, the second is nanosized fertilizers, manufactured on a nanoscale, and the third is nanoscale coated fertilizers, which are nanopolymer-wrapped fertilizers to contain regular size fertilizers [6]. This makes the nutrients available for a longer period compared to high-solubility fertilizers, which quickly release their nutrients [7], in response to environmental triggers or plant requirements [8].

In the use of fertilizers, it is necessary to investigate the way in which nanofertilizers are applied to satisfy the amount of nutrients required by plants [9], for their growth and improvement of productivity, making the release compatible with the absorption of nutrients with strategies efficient in their use [10]. The slow-release mechanism can be synchronized according to the growth and growth environment of the plants [9], by increasing the nutrient efficiency, the excessive use of fertilizers is reduced, which increases environmental safety [11]. The preparation of nanofertilizers can be done with organic materials, inorganic natural polymers, synthetics among others [12]. Clay is a carrier material that retains nutrients in its nanopores and is used to form structures that control the release of nutrients, called nanocomposites [13–16], which incorporate nanoparticles into their multiphase silicate matrices to improve the property of the material [17]. The matrix is ​​filled with nanometer-scale spaces with at least one dimension [18], and its natural structure generates a surface which is enabled to accept and release positive ions [20]. Therefore, nanostructured clays are considered nutrient carriers as they have ionic system that retain positive and negative ions that rely on them to neutralize [19].

Nitrogen is essential for proper plant growth, important for many structural, genetic and metabolic compounds in plant cells. The available uptake forms of inorganic nitrogen (NH_4_^+^ and NO_3_^−^) in the soil are less than 5% of the total nitrogen in the soil [21]. By applying inorganic and organic fertilizers, the nutritional condition of different agricultural systems can be maintained [22, 23]. However, nitrogen added to the soil due to harsh climatic conditions is partially lost to the solution in the form of denitrification, nitrate leaching and ammonia [24]. As a consequence of the rapid nutrient release from conventional fertilizers [25], macronutrients such as nitrogen are lost by 40–70% when applied to the soil, causing considerable loss of resources [4]. The ionic activity of clays, their ability to hold a wide range of ions and high ionic exchange capacity are considered suitable for slow nutrient release. In this sense, several studies have been carried out with nanostructures such as nanofertilizers, controlled release nitrogen fertilizer based on layered double hydroxides to evaluate nitrate release in water and soil [26], nitrate LDH to deliver nitrogen to the soil in a sustainable way [27], montmorillonite–urea [13] and in plant with bentonite nitrate [28]. In accordance with the previously mentioned, the objective of this study was to evaluate a nitrogen nanocomposite, its effect on leaching and N content of lettuce (*Lactuca sativa* L.) crop in soil columns.

## Materials and methods

### Fertilizer preparation

The nitrogenous nanocomposite was obtained at the Autonomous University of San Luis Potosí in the laboratories of the Faculty of Agronomy and Veterinary under the using the methodology proposed by Romero-Mendez et al. [28], consisting of modifying a bentonite-type clay through the sorption of a hexadecylamine cationic surfactant (HDA CH_3_ (CH_2_)15NH_2_, Aldrich Chemicals), to subsequently charge the nitrate ion (NO_3_^−^). The concentration of 110 mg g^−1^ of NO_3_^−^ used was determined by the difference of the final and initial concentration. The interlaminar spaces of the nanocomposite were shown in a range of 60–100 nm. For morphological characterization, a scanning electron microscope (SEM, UHR FEI HELIOS NANOLAB 600, USA) and a transmission electron microscope (model JEM 1230, JEOL, USA) were used to observe the size of the particles.

### Experiment setup

In the hydroponics greenhouse of the Faculty of Agronomy and Veterinary, an experiment with lettuce plants was established in April 2022 in a soil column system using a 9 × 5 m tunnel-type greenhouse. Lettuce seedlings of the Montemar variety were used, the soil columns consisted of PVC tubes of 11 cm in diameter and 20 cm height, and the column was filled with 2.37 kg to match the density of the soil (1.37 g cm^−3^). In the lower part of the column, a plastic mesh with opening holes of less than 1 mm was placed to contain the soil inside the column and not interrupt the flow of leachate, in addition to a container at the bottom to collect the drainage.

The lettuce seedlings used were the Montemar variety, which were planted in 200 cavity polystyrene trays, in sunshine mix 3 ® substrate, and transplanted into the soil of the columns when they had four true leaves. The characteristics of the soil are shown in Table 1.

**Table 1:** Characteristics of the soil used during the experiment to evaluate the effect of a nitrogenous nanocomposite on lettuce in soil columns

Determinations	Value
Bulk density	1.37 g cm^−3^
Field capacity	10.7%
Permanent wilting point	5.3%
Usable water	5.4%
Soil water at saturation	24%
Texture	Sandy loam
pH in water (1:2)	7.4
pH in saturation extract	7.9
CE in saturation Extract	0.5 mS cm^−1^
Total, carbonates	8.5%
Organic matter	1.3%
Inorganic nitrogen	6.1 kg Ha^−1^
Extractable phosphorus	71 kg Ha^−1^
Potassium	328 kg Ha^−1^

### Treatments

Fertilization treatments were evaluated in the soil columns with different proportions of nitrogen supplied with the nitrogenous nanocomposite. The dose 115-69-210 (N, P_2_O_5_, K_2_O) [29] the treatments evaluated was the dose of complementary nitrogen with nitrogenous nanofertilizer and conventional fertilization in a ratio of 25/0, 50/0, 75/0 and 100/0, 0/100, 25/75, 50/50 and 75/25 (Table 2).

**Table 2:** Treatments applied during the experiment to evaluate the effect of a nitrogenous nanocomposite on lettuce in soil columns

No.	Nitrogenous nanocomposite (%)	Conventional fertilizer (%)	Total units (%)	Treatment (%)
1	0	100	100	0/100
2	25	75	100	25/75
3	50	50	100	50/50
4	75	25	100	75/25
5	25	0	25	25/0
6	50	0	50	50/0
7	75	0	75	75/0
8	100	0	100	100/0

The commercial fertilizers that were used to apply the fertilization dose were: K_2_HPO_4_, K_2_SO_4_ and Ca (NO_3_^−^)_2_ + 4H_2_O, which were applied at the beginning and on the surface of the column as well as in the part corresponding to the NCN. Irrigation consisted of a total volume of 8100 mL distributed over the first 15 days in a volume of 100 mL in each column, the second 15 days 150 mL and the last 15 days 250 mL, in addition to adding 200 mL on days 15, 30 and 45 to obtain a leachate.

### Response variables

The variables determined every 15 days in the leached solution were electrical conductivity (EC) (Meter, Walfront, China), NO_3_^−^ K^+^ Ca^+2^ ion content (Laqua Twin Ionometer, Horiba, Japan), as well as the evolution of the SPAD unit variables (Soil Plant Analysis Development, SPAD-502 Plus Chlorophyll Meter 2900P, Spectrum Technologies, Illinois, USA), and the normalized difference vegetation index (NDVI, Green Seeker Trimble handheld crop sensor, California, USA). At the end of the experiment, the fresh weight was evaluated on a digital scale (Ohaus PAJ4102N Gold series, USA), leaf area (CID 202 Portable Leaf Area Meter, USA) and total dry biomass in a forced-air drying oven (Omron, Japan) at 72 °C, until constant weight was obtained on a digital scale (Ohaus PAJ4102N Gold series, USA), and the analysis of ions (NO_3_^−^, K^+^ and Ca^+2^) was carried out in the petiole cell extract [30] (Laqua Twin Ionometer, Horiba, Japan); the foliar N was determined by the Kjeldahl method [31],

### Experimental design and statistical analysis

The experiment was established in a completely randomized design, with 8 repetitions per treatment. The experimental unit consisted of a lettuce plant placed in a column of soil. The data obtained in the experiments were subjected to an analysis of variance, and the means of the treatments were compared using a Tukey test (*p* < 0.05), performed with SAS version 9.0.

## Results and discussion

**Fig. 1:**
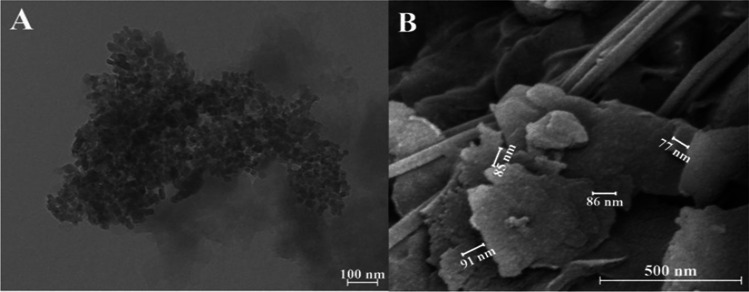
Transmission electron microscopy (TEM) image, magnitude 100 nm (**a**), scanning electron microscopy (SEM) image, magnitude 500 nm (**b**) of a nitrogenous nanocomposite

The size and distribution of the particles of the synthesized NCN were investigated by transmission electron microscope (TEM) at the energy of 100 kV, shown in Fig. 1a The TEM image showed the uniform distribution of particles with size, where most of the particles were 20 nm. Clay nanofertilizers such as bentonite retain nutrients in multiphase silicate matrices that are incorporated with nanoparticles to improve the property of the material. In this case, the fertilizing material is inside the bentonite sheets and their space is less than 100 nm, as shown in Fig. 1b. These materials create a crystalline three-dimensional structure of cavities, channels and/or pores at a nanometer scale. Its natural structure generates a surface that can retain water, as well as the exchange of cations [20], which are composed of a matrix filled with reinforcements with at least one dimension on the nanometer scale [18].

### Leaf area

In Table 3, leaf area shows that most of the treatments were statistically similar with average values of 2850 cm^2^. Only the 25/0 treatment decreased 23% with respect to the control treatment. However, it was statistically equal to treatment 50/0 and 75/0, which in turn were equal to the other treatments. The 100/0 treatment statistically generated the same amount of area as the control, which indicates that the 100% NCN application produces the same leaf area as conventional fertilizers. Insufficient amount of nitrogen can limit plant growth by having lower N availability at lower doses [32]. However, the leaf area increased by 31% when applying an NPK nanofertilizer with half the recommended dose in lettuce [33]. The synchronization of the release of nitrogen in the slow-release fertilizer with the lettuce crop is important because if not released when the plant requires it, the leaf area decreases by absorbing fewer nutrients [34]. In this sense, applying nanofertilizers in doses of 50, 75 and 100% without conventional fertilizer can be a viable alternative for the growth of lettuce since the slow release does not limit the leaf area.

**Table 3:** Conventional fertilization/nitrogenous nanocomposites on growth variables in the evaluation of the effect of a nitrogenous nanocomposite on lettuce in soil columns

Treatments	Fresh weight (g)	Leaf area (cm^2^)	Dry biomass (g)		
			Leaves	Root	Total
0/100	186.74 a	2884 a	11.23 a	6.44 ab	17.67 a
25/75	179.86 ab	2901 a	11.26 a	7.50 a	18.76 a
50/50	179.00 ab	2917 a	10.89 a	6.77 ab	17.67 a
75/25	179.04 ab	2901 a	10.41 a	7.15 a	17.56 a
25/0	138.53 b	2201 b	5.79 c	4.67 b	9.16 c
50/0	154.16 ab	2647 ab	6.88 bc	5.85 ab	12.74 bc
75/0	149.44 ab	2822 ab	9.25 ab	7.25 a	16.50 ab
100/0	179.33 ab	2896 a	10.10 a	6.12 ab	16.21 ab
DMS	47.84	354.58	3.18	2.1	4.11
CV	18.06	8.01	21.32	20.67	16.39

Different letters in the treatments indicate significant differences Tukey; (*P* < 0.05)

### Fresh weight

For lettuce fresh weight (Table 3), the 25/0 treatment reduced fresh weight by 25.8% compared to the control treatment 0/100, the other treatments had similar values to the control and 25/0 with an average of 160 g fresh weight. Nofal [33] mentioned that the use of NPK nanofertilizers increased the fresh weight of lettuce by 24% using half the recommended dose, and this could be due to the retention and slow release of ions according to crop demand. In our case, the doses with 25/0 nanofertilizers decreasing the fresh weight could be due to the fact that the interlaminar spaces do not carry enough nitrogenous material to satisfy the demand of the lettuce crop.

### Dry biomass

The dry biomass of the leaves presented significant differences between treatments in Table 3. The 50/0 and 25/0 treatments reduced dry biomass 38.7% and 48.4%, respectively, compared to the control treatment, while the other treatments were equal to the control. The same trend was shown in the dry biomass of the roots, wherein the 25/0 treatment decreased 27.5% with respect to the control 0/100. The other treatments were similar with an average of 6.7 gr. For total dry biomass, there were significant differences for the same treatments 25/0 and 50/0, decreasing 48 and 28%, respectively. A lettuce crop evaluated with a nanonitrogen fertilizer in soil 25% conventional fertilizer and 75% nanoparticles in two crop cycles increased 123 to 159% in plant weight [35]. Similarly, the dry biomass in lettuce increased when applying an NPK nanofertilizer with half the recommended dose [33]. The application of nanoshaped nitrogen increases growth, yield, by improving protein content and uptake of other essential nutrients [36]. The addition of the clay mineral struvite as a slow-release fertilizer to the lettuce crop significantly increased the mean fresh and dry weight of the lettuce compared to the control [37]. When slow-release and fast-release nitrogen fertilizers were evaluated at doses of 0, 60, 90 and 120 kg N ha^−1^ applied to lettuce plants, it was found that the increase in the level of nitrogen fertilizer is associated with an increase in the fresh and dry weight of the plants. However, the slow-release fertilizer with the highest dose was greater in fresh and dry biomass [38]. In this sense, nitrogen content in the NCN 25 and 50 doses without traditional fertilizer was not sufficient for the plant to grow properly, and this negatively affected total dry biomass.

### Ion content in petiole cell extract

**Fig. 2:**
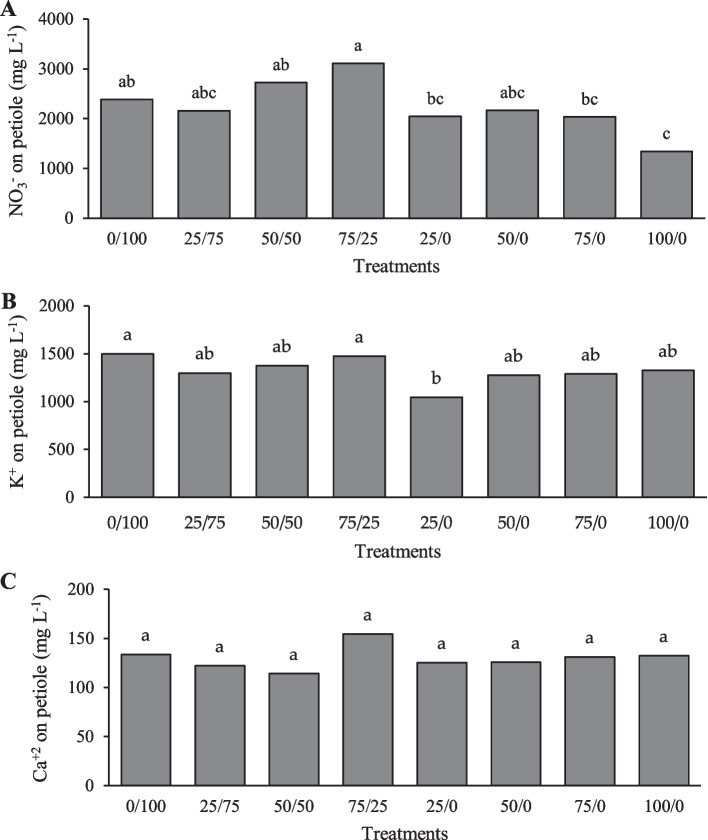
Content of **A** nitrate, **B** potassium and **C** calcium in the petiole cell extract in the evaluation of the effect of a nitrogenous nanocomposite in lettuce in soil columns. Different letters in the treatments indicate significant differences Tukey; (*P* < 0.05)

The nitrate content in the petiole cell extract showed significant differences between the treatments as shown in Fig. 2A. The highest value was presented with the 75/25 treatment (3113 mg L^−1^), which was 34% higher than the 25/0, 75/0 treatments and 56% higher than the 100/0 treatment. In crops such as raspberry, melon and pepper, they found negative correlations indicating that higher application rates do not reflect higher assimilations, due to the combination of high mineral applications with periods of low plant demand [39]. In this sense, Llanderal [40] found that the concentration of nutrients in the petiole cell extract is a function of the level of ion demand for some physiological processes, such as absorption, bioassimilation and storage of nutrients. In addition to the climatic conditions during the growing seasons and the fertilizer used, as mentioned by Lara-Izaguirre [41], in lettuce with different ratios of NO_3_^−^/NH_4_^+^, 5431 (mg L^−1^) of NO_3_^−^ was found in the summer and 2416 (mg L^−1^) in autumn. On the other hand, in the soil the decrease in the ionic solution is due to the fact that the plants showed a greater assimilation in certain phenological stages or to the lower depth of leaching [42]. In this case, the lower content of NO_3_^−^ in the cell extract of the petiole in the lettuce crop could be that the plant in the final stage had lower demands for this element with a higher concentration of NCN.

The potassium ion content in the analysis of the petiole was similar between treatments as shown in Fig. 2B, except for treatment 25/0 which generated 30% less than the control treatment. The average of the other treatments was 1360 mg L^−1^. In petiole calcium ion, there were no significant differences as shown in Fig. 2C. The average of the treatments was 130 mg L^−1^. However, the 75/25 treatment was slightly higher than the other treatments with 155 mg L^−1^. Benavides-Mendoza [39] conducted field studies to determine the ranges of the petiole cell extract in lettuce, and the ionic concentration ranges in the petiole cell extract provide an approximation to the ranges of nutritional sufficiency for K 2950–3325 mg L^−1^ and 104–125 mg L^−1^ for Ca^+2^.

### SPAD units

**Fig. 3:**
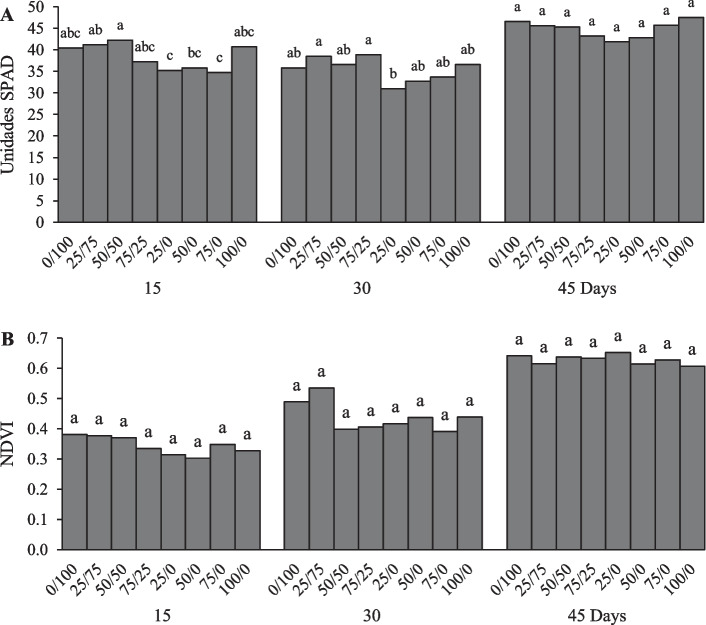
SPAD units (**A**), NDVI (**B**) measured at 15, 30 and 45 days in the evaluation of the effect of a nitrogenous nanocomposite on lettuce in soil columns. Different letters in the treatments indicate significant differences, Tukey (*P* < 0.05)

Figure 3A shows the different measurements that were taken during the experiment. Significant differences were shown at 15 and 30 days of evaluation: In the first, the 25/0 and 75/0 treatments decreased 12.6 and 13.8% compared to the control treatment, and the other treatments were the same as the control. On day 30, the treatment that decreased with respect to the control treatment was 25/0 with 13%, while at 45 days, and there were no significant differences between treatments Fig. 3A, which had an average value of 44.8. The SPAD is used to estimate the concentration of N in lettuce plants [43], and at higher doses of N, the higher the photosynthetic efficiency of the plant, the higher the SPAD units [44]. The effect of SPAD in lettuce does not vary in relation to the doses of NO_3_^−^/NH_4_^+^, but it does vary when cultivating in different cycles: values of 39.38 in autumn and 29.58 in summer [41]. Drip irrigation makes the use of nanofertilizers in lettuce more efficient by increasing 25% of SPAD units (38 to 44) compared to the control applied to the soil (30–35) [35]. In this case, neither with conventional fertilizer nor with NCN the doses of N showed a difference in the values at the end of the harvest, which suggests that the concentration of N applied reflected in the SPAD was similar.

### Normalized difference vegetation index (NDVI)

The NDVI at the different evaluation times showed no significant differences between treatments as shown in Fig. 3B. However, the increase can be observed due to the measurement time, with an average value at 15 days of 0.34, at 30 days it increased to 0.44, and at 45 days, it reached mean values of 0.63. Therefore, according to Ashraf [45] values close to 1, the index describes a stronger vegetative growth; in this case, the applied doses did not promote any stress with the NVDI. Galieni [46] mentioned that lettuce in growing seasons has no differences (0.82 to 0.85), and in treatments without N, the NVDI values are reduced. In carrot, slow-release nitrogen fertilizers were evaluated for a period of 3 years, the treatments did not affect the NDVI readings, they were in the ranges of 0.76 and 0.80, and it was possible to determine that the slow-release fertilizers require only one application in comparison with the control with two applications. This reduces environmental impacts, less leaching, due to the release of N over a longer period compared to traditional N source products [47]. Nitrogen status was evaluated in geraniums with controlled release fertilizer, with different doses from 0 to 16 g in greenhouse-grown plants. The NDVI values were higher: 0.78 for the plants that received application of N fertilizer and 0.51 for the plants that did not receive fertilizer. As the plants grew, the NDVI was able to differentiate between the fertilizer doses where the treatments were higher, and the differences can be attributed to the release rates, distribution and N content [48]. In this sense, the NDVI allows monitoring crop health during the growing season to confirm whether preplant N application of N fertilizer was sufficient to meet crop needs [47]. Since this experiment did not show significant differences in the NVDI, we can mention that the amount of N applied in the different treatments with conventional fertilizer and with nanocomposite was in adequate amounts for lettuce, as the normalized difference index was similar for all; therefore, the plant did not show any type of stress in any treatment that could be differentiated with the NVDI.

### Analysis of the leached solution

#### Electrical conductivity

**Fig. 4:**
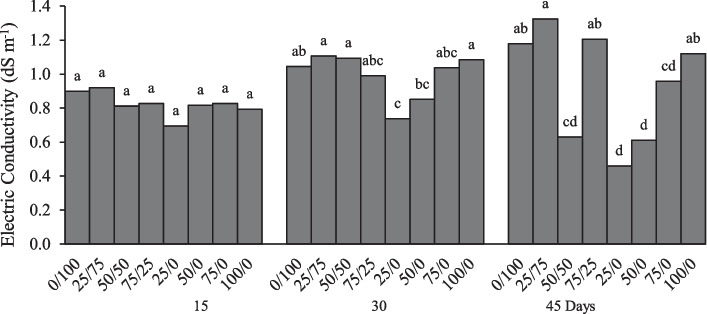
Electrical conductivity (dS m^−1^) of the leached solutions measured at 15, 30 and 45 days in the evaluation of the effect of a nitrogenous nanocomposite on lettuce in soil columns. Different letters in the treatments indicate significant differences, Tukey (*P* < 0.05)

At 15 days, the EC of the leached solutions did not show significant differences between treatments as shown Fig. 4, the average value was 0.69 dS m^−1^, while at 30 days the 25/0 treatment had the lowest value, the same for the day 45 in addition to the 50/0 treatment. The EC of the leachate during the first weeks of the study is associated with the nutrient release from the controlled release fertilizer and with the soluble salts leached from the substrate [49], in addition to the fact that the plants absorb them much easier [50]. The conductivity increases due to the release of nutrients from the fertilizer [51]. In this sense, we can observe that the EC was higher in this work, especially in the treatments where both nano- and conventional fertilizers were applied (0/100, 25/75, 75/25 and 100/0).

#### NO_3_^−^ K^+^ and Ca^+2^ leaching

**Fig. 5:**
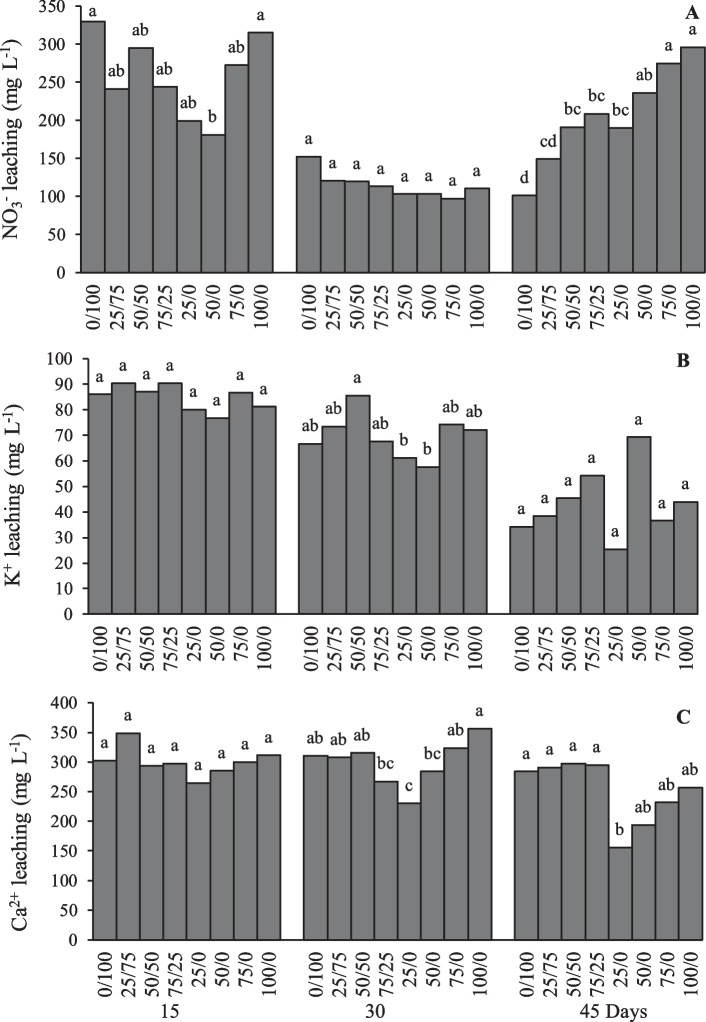
A NO_3_^−^, **B** K^+^ and **C** Ca^2+^ leaching in the evaluation of the effect of a nitrogenous nanocomposite on lettuce in soil columns. Different letters in the treatments indicate significant differences, Tukey (*P* < 0.05)

In Fig. 5A, the nitrate content in the leachate after 15 days showed significant differences, where the 50/0 treatment reduced 45% compared to the control treatment. By day 30, the content decreased by half; however, there were no differences between treatments. On day 45, the 75/0 and 100/0 treatments were statistically higher than all the treatments with traditional fertilizer application and the lowest dose of NNC was found on 25/0. In an evaluation in sugarcane, the application of doses of urea and nitrogen chelated nanofertilizers showed that the highest nitrate leaching (699.0 mg L^−1^) belongs to the highest level of urea fertilizer and to the lowest level NNC applied (183.0 mg L^−1^). Increasing nitrogen levels in all fertilizer treatments increased soil nitrate concentration and nitrate leaching [52]. In soil column, the application of multiple N  pellets of lignite urea, a delayed release N fertilizer material, can potentially reduce N losses and, at the same time, add N to the soil profile. The lower leaching loss of mineral N from the pellets containing the higher amount of lignite emphasizes the role in N retention. The higher nutrient retention may occur through the formation of organo-mineral complexes that are more stable and less are likely to be lost by leaching or volatilization [53]. The yield with nanonitrogenous fertilizer was higher when increasing the amount of fertilizer, given the low leaching rate that slow- release fertilizers have [54]. Less nutrient leaching with the use of slow-release nanofertilizers can reduce soil and water pollution.

For the K^+^ ion in the first 15 days of cultivation, the treatments did not show significant differences, as shown in Fig. 5B, showing average values of 85 mg L^−1^; likewise, in the following 30 days, the average decreased to 70 mg L^−1^ with no differences between treatments, except for 25/0 and 50/0 treatments with a value of 60 mg L^−1^. Finally, on day 45 the leaching remained without differences between treatments with a decrease of 40 mg L^−1^. In the calcium leaching shown in Fig. 5C, the average concentrations were 300, 270 and 250 mg L^−1^ for days 145, 30 and 45 days, respectively, without statistically affecting the treatments, except on day 45 with 150 mg L^−1^ in treatment 25/0. In an evaluation of nutrient release by slow-release compressed NPK fertilizers in soil columns, Fernandez-Sanjurjo [55] obtained concentrations of leached K^+^ and Ca^+2^ ions of 20–40 and 20–50 (mg L^−1^), respectively. In our case, the leaching of both ions decreased with time, indicating normal nutrient uptake.

#### Nitrogen content in leaf

**Fig. 6:**
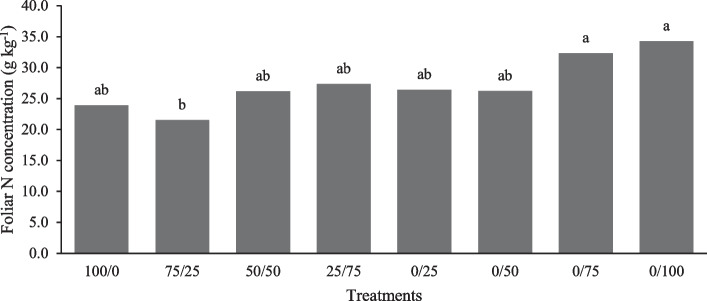
Nitrogen concentration in lettuce leaves in the evaluation of the effect of a nitrogenous nanocomposite on lettuce in soil columns. Different letters in the treatments indicate significant differences, Tukey (*P* < 0.05)

The application of NCN in different combinations with conventional fertilizers significantly affected the concentration of nitrogen in the lettuce plant, with a higher content in the 0/75 and 0/100 treatment by 33% compared to the 75/25 treatment, and the rest of the treatments did not present differences, as shown in Fig. 6. However, Mills and Jones [56] mentioned that lettuce values are in a sufficiency range of 2.5 to 4%, where most in this work is above 2.5% except for 100/0 and 75/25 treatments. In an experiment with the cultivation of lettuce with three treatments: urea, starch–urea mixture and corn starch carbamate (slow-release fertilizer), lettuce nitrogen content was not significantly different between treatment groups, as all treatments absorbed nitrogen at similar levels, and starch carbamate can be used as a slow-release fertilizer due to its low solubility and release rate and mineralization [57]. In lettuce plants, the nutrient uptake was evaluated with the application of nanonitrogen under different irrigation and essential regimes on the lettuce plant with different combinations of nanonitrogen forms and in bulk with surface irrigation method and by drip. The maximum N contents (5.15 to 5.87%) were detected by applying N at a rate of 75% in massive drip irrigation and 25% in nanoform through foliar application in both seasons [35].

The data show that, under the specific conditions of the study, total substitution and up to 25% reduction of mineral N by basal application of nanofertilizers are the best strategy to minimize the use of conventional fertilizers in lettuce cultivation without compromising yield. However, the result may depend to a large extent on the soils used and their physical, chemical and biological composition. Therefore, it is recommended that future studies should evaluate other types of soils, more doses of nanofertilizers, lettuce varieties and application times before reaching a final conclusion.

## Conclusions

The use of doses with nitrogenous nanocomposite in the soil columns released fertilizer in similar amounts to the control with conventional fertilizer that did not affect the growth of the lettuce plant. Doses with 75 and 100% NCN have a similar effect on the lettuce plant growth. The nitrogen concentration in the leaves is within the optimal values. However, the release of the nitrogenous nanocomposite increased the number of nitrates in the leachate.

The application of nitrogen to the soil in the form of a nitrogenous nanocomposite can be an option to replace the use of conventional fertilizers, since the 50 and 75 treatments require less quantity than the control treatment. However, the same results are obtained; that is, the same is produced with less quantity and reduces the nitrogen in the leachates. The application of nitrogenous nanocomposite not only matches the growth characteristics of the crop, but also allows the use of lower doses than those recommended.

## Data Availability

The datasets generated during and/or analyzed during the current study are available from the corresponding author on reasonable request.

## References

[1] Kalia A, Sharma SP, Kaur H, Kaur H. Novel nanocomposite-based controlled-release fertilizer and pesticide formulations: prospects and challenges. In: Multifunctional hybrid nanomaterials for sustainable agri-food and ecosystems, pp. 99–134 (2020).

[2] Stanley N, Mahanty B (2020). Preparation and characterization of biogenic CaCO_3_-reinforced polyvinyl alcohol–alginate hydrogel as controlled-release urea formulation. Polym Bull.

[3] Rashid M, Hussain Q, Khan KS, Alwabel MI, Hayat R, Akmal M, Alvi S (2021). Carbon-based slow-release fertilizers for efficient nutrient management: synthesis, applications, and future research needs. J Soil Sci Plant Nutr.

[4] Zulfiqar F, Navarro M, Ashraf M, Akram NA, Munné-Bosch S (2019). Nanofertilizer use for sustainable agriculture: advantages and limitations. Plant Sci.

[5] Ndaba B, Roopnarain A, Haripriya R, Maaza M (2022). Biosynthesized metallic nanoparticles as fertilizers: an emerging precision agriculture strategy. J Integr Agric.

[6] Shao C, Zhao H, Wang P (2022). Recent development in functional nanomaterials for sustainable and smart agricultural chemical technologies. Nano Converg.

[7] Shaji H, Chandran V, Mathew L. Organic fertilizers as a route to controlled release of nutrients. In: controlled release fertilizers for sustainable agriculture. 2021 pp. 231–245. Academic Press. 10.1016/B978-0-12-819555-0.00013-3

[8] Wang P, Lombi E, Zhao FJ, Kopittke PM (2016). Nanotechnology: a new opportunity in plant sciences. Trends Plant Sci.

[9] Al-Mamun MR, Hasan MR, Ahommed MS, Bacchu MS, Ali MR, Khan MZH (2021). Nanofertilizers towards sustainable agriculture and environment. Environ Technol Innov.

[10] Shang Y, Hasan MK, Ahammed GJ, Li M, Yin H, Zhou J (2019). Applications of nanotechnology in plant growth and crop protection: a review. Mol.

[11] Panpatte DG, Jhala YK (2019). Nanotechnology for agriculture: crop production & protection.

[12] Servin A, Elmer W, Mukherjee A, la Torre-Roche D, Hamdi H, White JC, Bindraban P, Dimkpa C (2015). A review of the use of engineered nanomaterials to suppress plant disease and enhance crop yield. J Nanopart Res.

[13] Golbashy M, Sabahi H, Allahdadi I, Nazokdast H, Hosseini M (2016). Synthesis the montmorillonite-pomegranate (*Punicagranatum* L.) peel polyphenols nanostructure as a drug delivery vehicle. Biomed Pharmacol.

[14] Kottegoda N, Sandaruwan C, Priyadarshana G, Siriwardhana A, Rathnayake UA, Berugoda Arachchige DM, Amaratunga GA (2017). Urea-hydroxyapatite nanohybrids for slow release of nitrogen. ACS Nano.

[15] Borges R, Wypych F, Petit E, Forano C, Prevot V (2019). Potential sustainable slow-release fertilizers obtained by mechanochemical activation of MgAl and MgFe layered double hydroxides and K_2_HPO_4_. J Nanomater.

[16] Tarafder C, Daizy M, Alam MM, Ali MR, Islam MJ, Islam R, Khan MZH (2020). Formulation of a hybrid nanofertilizer for slow and sustainable release of micronutrients. ACS Omega.

[17] Naseem F, Zhi Y, Farrukh MA, Hussain F, Yin Z (2020). Mesoporous ZnAl_2_Si_10_O_24_ nanofertilizers enable high yield of *Oryza sativa* L. Sci Rep.

[18] Saadi S, Nazari B (2019). Recent developments and applications of nanocomposites in solar cells: a review. J Compos Compd.

[19] Lazaratou CV, Vayenas DV, Papoulis D (2020). The role of clays, clay minerals and clay-based materials for nitrate removal from water systems: a review. Appl Clay Sci.

[20] Bernardi AC, Monte MBDM, Paiva PRP, Werneck CG, Haim PG, Barros FDS (2010). Dry matter production and nutrient accumulation after successive crops of lettuce, tomato, rice, and andropogongrass in a substrate with zeolite. Rev Bras Cienc Solo.

[21] Brady NC, Weil RR, Brady NC, Weil RR (2008). Soil Colloids: Seat of Soil Chemical and Physical Acidity. The Nature and Properties of Soils.

[22] Ginting D, Kessavalou A, Eghball B, Doran JW (2003). Greenhouse gas emissions and soil indicators four years after manure compost applications. J Environ Qual.

[23] Watts DB, Torbert HA, Prior SA, Huluka G (2010). Long-term tillage and poultry litter impacts soil carbon and nitrogen mineralization and fertility. Soil Sci Soc Am J.

[24] Cameron KC, Di HJ, Moir JL (2013). Nitrogen losses from the soil/plant system: a review. Ann Appl Biol.

[25] Khajavi-Shojaei S, Moezzi A, Norouzi MM, Taghavi M (2020). Synthesis modified biochar-based slow-release nitrogen fertilizer increases nitrogen use efficiency and corn (*Zea mays* L.) growth. Biomass Convers Biorefinery.

[26] Ureña-Amate MD, Boutarbouch ND, del Mar Socias-Viciana M, González-Pradas E (2011). Controlled release of nitrate from hydrotalcite modified formulations. Appl Clay Sci.

[27] Berber MR, Hafez IH, Minagawa K, Mori T (2014). A sustained controlled release formulation of soil nitrogen based on nitrate-layered double hydroxide nanoparticle material. J Soils Sediments.

[28] Romero-Méndez MJ, Rojas-Velázquez ÁN, Mireles JLL, Flores PED, Reza JLW (2019). Efecto de un fertilizante nitrogenado a base de bentonita modificada y tensoactivo HDTMA en el crecimiento de lechuga (*Lactuca sativa* L.) hidropónica. Agrociencia.

[29] Velásquez P, Ruíz H, Chaves G, Luna C (2014). Productividad de lechuga *Lactuca sativa* en condiciones de macrotúnel en suelo Vitric haplustands. Rev Mexicana Cienc Agric.

[30] Damian Nava A, Arellano Roque L, Hernandez Castro E, Palemon Alberto F, Cruz Lagunas B, Vargas Alvarez D. Effect of Organic Nutrition in the Nursery Growth and Nutrimental Content of Native Avocados of Ometepec, Guerrero, Mexico; 2017.

[31] Lynch JM, Barbano DM (1999). Kjeldahl nitrogen analysis as a reference method for protein determination in dairy products. J AOAC Int.

[32] Marschner H, Marschner P. Marschner’s Mineral nutrition of higher plants. 3rd ed. London: Elsevier/London: Academic Press; 2012.

[33] Nofal AS, Ashmawi AE, Mohammed AA, El-Abd MT, Helaly AA (2021). Effect of soil application of nano NPK fertilizers on growth, productivity and quality of Lettuce (*Lactuca sativa*). Azhar J Agric Res.

[34] Okyay G, Karagöz S, Ulaş A, Özen İ (2020). Efficiency of an agrotextile surface structure possessing fertilizer and water management coupled with mulching property in romaine lettuce growth trials. J Text Inst.

[35] Sharaf-Eldin MA, Elsawy MB, Eisa MY, El-Ramady H, Usman M, Zia-ur-Rehman M (2020). Application of nano-nitrogen fertilizers to enhance nitrogen efficiency forlettuce growth under different irrigation regimes. Pak J Agric Sci.

[36] Zahedi SM, Karimi M, Teixeira da Silva JA (2020). The use of nanotechnology to increase quality and yield of fruit crops. J Sci Food Agric.

[37] Ryu HD, Lim CS, Kim YK, Kim KY, Lee SI (2012). Recovery of struvite obtained from semiconductor wastewater and reuse as a slow-release fertilizer. Environ Eng Sci.

[38] Awaad MS, Badr RA, Badr MA, Abd-elrahman AH (2016). Effects of different nitrogen and potassium sources on lettuce (*Lactuca sativa* L.) yield in a sandy soil. Eurasian J. Soil Sci..

[39] Benavides-Mendoza A, Alba-Romenus KD, Francisco-Francisco N (2021). Relation between soil solution composition and petiole cellular extract of crops in western Mexico. Terra Latinoam.

[40] Llanderal A, García-Caparrós P, Pérez-Alonso J, Contreras JI, Segura ML, Reca J, Lao MT (2020). Approach to petiole sap nutritional diagnosis method by empirical model based on climatic and growth parameters. Agronomy.

[41] Lara-Izaguirre AY, Rojas-Velázquez AN, Romero-Méndez MJ, Ramírez-Tobías HM, Cruz-Crespo E, Alcalá-Jáuregui JA, Loredo-Ostí C (2019). Growth and no3-accumulation in hydroponic lettuce with nitrate/ammonium ratios in two cultivation seasons. Rev Fitotec Mex.

[42] Komosa A, Roszyk J, Mieloch M (2017). Content of nutrients in soils of highbush blueberry (*Vaccinium corymbosum* L.) plantations in Poland in a long-term study. J Elementol.

[43] Mendoza-Tafolla RO, Juarez-Lopez P, Ontiveros-Capurata RE, Sandoval-Villa M, Iran AT, Alejo-Santiago G (2019). Estimating nitrogen and chlorophyll status of romaine lettuce using SPAD and at LEAF readings. Not Bot Horti Agrobot Cluj Napoca.

[44] Cunha ARD, Katz I, Sousa AP, Martínez Uribe RA (2015). Índice SPAD en el crecimiento y desarrollo de plantas de lisianthus en función de diferentes dosis de nitrógeno en ambiente protegido. Idesia (Arica).

[45] Ashraf A, Hussain I, Ahmad MM, Iqbal MB, Ali M, Hussain Q (2016). Crop growth monitoring using green seeker technology—a case of NARC Field Station in Pothwar Region: Green Seeker Technology. Proc Pak Acad Sci B Life Environ Sci.

[46] Galieni A, Stagnari F, Speca S, Pisante M (2016). Leaf traits as indicators of limiting growing conditions for lettuce (*Lactuca sativa*). Ann Appl Biol.

[47] Sanderson KR, Fillmore SAE (2012). Slow-release nitrogen fertilizer in carrot production on Prince Edward Island. Can J Plant Sci.

[48] Dunn BL, Shrestha A, Goad C (2015). Determining nitrogen fertility status using optical sensors in geranium with controlled release fertilizer. J Appl Hortic.

[49] Merhaut DJ, Blythe EK, Newman JP, Albano JP (2006). Nutrient release from controlled-release fertilizers in acid substrate in a greenhouse environment: I. Leachate electrical conductivity, pH, and nitrogen, phosphorus, and potassium concentrations. HortScience.

[50] Andiru GA, Pasian CC, Frantz JM (2015). Effects of controlled-release fertilizer placement on nutrient leaching and growth of bedding impatiens. J Environ Hortic.

[51] Zanin G, Maucieri C, Dal Ferro N, Bortolini L, Borin M (2020). Evaluating a controlled-release fertilizer for plant establishment in floating elements for bioretention ponds. Agronomy.

[52] Alimohammadi M, Panahpour E, Naseri A (2020). Assessing the effects of urea and nano-nitrogen chelate fertilizers on sugarcane yield and dynamic of nitrate in soil. Soil Sci Plant Nutr.

[53] Saha BK, Rose MT, Wong VN, Cavagnaro TR, Patti AF (2018). Nitrogen dynamics in soil fertilized with slow-release brown coal-urea fertilizers. Sci Rep.

[54] Zareabyaneh H, Bayatvarkeshi M (2015). Effects of slow-release fertilizers on nitrate leaching, its distribution in soil profile, N-use efficiency, and yield in potato crop. Environ Earth Sci.

[55] Fernández-Sanjurjo MJ, Álvarez-Rodríguez E, Núñez-Delgado A, Fernández-Marcos ML, Romar-Gasalla A (2014). Nitrogen, phosphorus, potassium, calcium and magnesium release from two compressed fertilizers: column experiments. Solid Earth.

[56] Mills HA, Benton JJ. Plant analysis handbook II: a practical preparation, analysis, and interpretation guide (No. 631.42/J76). MicroMacro Publishing, Athens. 1996.

[57] Kim DH, Kang YJ, Choi JJ, Yun SI (2020). Lettuce growth and nitrogen loss in soil treated with corn starch carbamate produced using urea. Korean J Soil Sci.

